# Ribosome inactivation regulates translation elongation in neurons

**DOI:** 10.1016/j.jbc.2024.105648

**Published:** 2024-01-12

**Authors:** Bastian Popper, Martina Bürkle, Giuliana Ciccopiedi, Marta Marchioretto, Ignasi Forné, Axel Imhof, Tobias Straub, Gabriella Viero, Magdalena Götz, Rico Schieweck

**Affiliations:** 1 Core Facility Animal Models, Biomedical Center (BMC), LMU Munich, Munich, Germany; 2 Department of Physiological Genomics, Biomedical Center (BMC), LMU Munich, Munich, Germany; 3 Department for Cell Biology & Anatomy, Biomedical Center (BMC), LMU Munich, Munich, Germany; 4 Graduate School of Systemic Neurosciences, LMU Munich, Munich, Germany; 5 Institute of Biophysics, National Research Council (CNR) Unit at Trento, Povo, Italy; 6 Protein Analysis Unit, Department for Molecular Biology, Biomedical Center (BMC), LMU Munich, Munich, Germany; 7 Bioinformatics Core Facility, Department of Molecular Biology, Biomedical Center (BMC), LMU Munich, Munich, Germany; 8 Institute of Stem Cell Research, Helmholtz Center Munich, German Research Center for Environmental Health, Munich, Germany; 9 SYNERGY, Excellence Cluster of Systems Neurology, Biomedical Center (BMC), LMU Munich, Munich, Germany

**Keywords:** ribosome speed, stem cells, astrocytes, neurons, polysome profiling, neuronal stimulation

## Abstract

Cellular plasticity is crucial for adapting to ever-changing stimuli. As a result, cells consistently reshape their translatome, and, consequently, their proteome. The control of translational activity has been thoroughly examined at the stage of translation initiation. However, the regulation of ribosome speed in cells is widely unknown. In this study, we utilized a timed ribosome runoff approach, along with proteomics and transmission electron microscopy, to investigate global translation kinetics in cells. We found that ribosome speeds vary among various cell types, such as astrocytes, induced pluripotent human stem cells, human neural stem cells, and human and rat neurons. Of all cell types studied, mature cortical neurons exhibit the highest rate of translation. This finding is particularly remarkable because mature cortical neurons express the eukaryotic elongation factor 2 (eEF2) at lower levels than other cell types. Neurons solve this conundrum by inactivating a fraction of their ribosomes. As a result, the increase in eEF2 levels leads to a reduction of inactive ribosomes and an enhancement of active ones. Processes that alter the demand for active ribosomes, like neuronal excitation, cause increased inactivation of redundant ribosomes in an eEF2-dependent manner. Our data suggest a novel regulatory mechanism in which neurons dynamically inactivate ribosomes to facilitate translational remodeling. These findings have important implications for developmental brain disorders characterized by, among other things, aberrant translation.

Translation is a multicomponent and multistep process that is strictly controlled from the initiation stage through elongation and termination (1, 2). Regulators, including RNA-binding proteins (RBPs) (3), transfer RNAs (tRNAs) (4, 5), codon usage (6), and RNA secondary structures (7), influence the efficiency of these steps. This control leads to distinct translational efficiencies of transcripts, which ultimately affect the levels of encoded proteins.

The key player in translation is the ribosome. While historically viewed as a molecular machine that reads mRNA to produce proteins, it is now recognized as a direct and active translation regulator (8). Therefore, it acts as a landing platform for other proteins such as RBPs or regulates translation through its expression levels (9, 10, 11). In both cases, ribosomes impact the translation of specific transcripts rather than the entire translatome (12, 13, 14). Thus, it has been hypothesized that specialized ribosomes exist that regulate the translation of subsets of mRNAs in a similar manner to RBPs (8, 15, 16). These posttranscriptional gene regulation mechanisms act together, shaping the cellular proteome and balancing global changes in gene expression (3, 17, 18, 19, 20).

Due to neurons' complex three-dimensional shape and their integration of multiple external stimuli, a precise regulation of their translatome in a spatiotemporal manner is required (21). The initiation phase is primarily regulated to select transcripts for translation (1). Additionally, elongation plays a pivotal role in the translational activity of specific transcripts and the entire translatome (4, 22, 23). The speed of ribosomes is determined by various factors, including tRNA concentrations (6), RBPs (3), RNA secondary structures (7, 24), and ribosome-associated proteins (25, 26, 27). Additionally, eukaryotic elongation factors 1 and 2 (eEF1, 2) play a crucial role in elongation, with eEF2 facilitating the translocation of the ribosome (28). Emerging evidence suggests that elongation speed varies among transcripts (29) and across different tissues (30). The adaptation of ribosome speed might be crucial for proteins whose folding and/or assembly into complexes is regulated cotranslationally such as the Cystic fibrosis transmembrane conductance regulator (4, 22, 31, 32, 33, 34, 35). For example, the codon optimality can regulate ribosome speed and protein folding and/or interaction (6, 32, 35, 36, 37). Pioneer studies have demonstrated that mutations in mRNAs (33, 34) or tRNAs (4, 38, 39, 40) can lead to protein misfolding, degradation, and ultimately, devastating diseases (4, 38, 39, 41). Since these mutations can alter ribosome speed, it is tempting to speculate that misregulated ribosome elongation is, at least partially, responsible for these phenotypes. Accordingly, in-depth understanding of the mechanisms underlying ribosome elongation is crucial to understanding its impact on protein homeostasis.

Here, we set out to investigate ribosome kinetics in different cell types to determine whether elongation is controlled in a cell type-specific manner. Our findings demonstrate that ribosome speed rates vary between different cellular identities including human embryonic kidney (HEK) cells, (neural) stem cells, astrocytes, and neurons. Furthermore, during neuronal development, ribosomes switch from a slow to a fast elongation rate (42). In this context, we found that mature neurons elongate the fastest of the cell types we have studied. This finding was surprising as these cells express eEF2 at lowest levels compared to other cell types. To resolve this conundrum, nerve cells inactivate polyribosomes in the cytosol. Ribosome inactivation is governed by eEF2 levels, where an increase in eEF2 reduces the number of inactive ribosomes. Additionally, processes like neuronal excitation that alter the demand for active ribosomes increases ribosome silencing in an eEF2 dependent manner. Based on our findings, we suggest a dual function for eEF2 in regulating ribosome elongation and inactivation. Thereby, lower levels of eEF2 lead to ribosome inactivation whereas higher levels results in active ribosomes. This mechanism helps to balance translational activity in neurons and may be crucial for translational plasticity.

## Results

### Polysome kinetics to study translation speed

**Figure 1:**
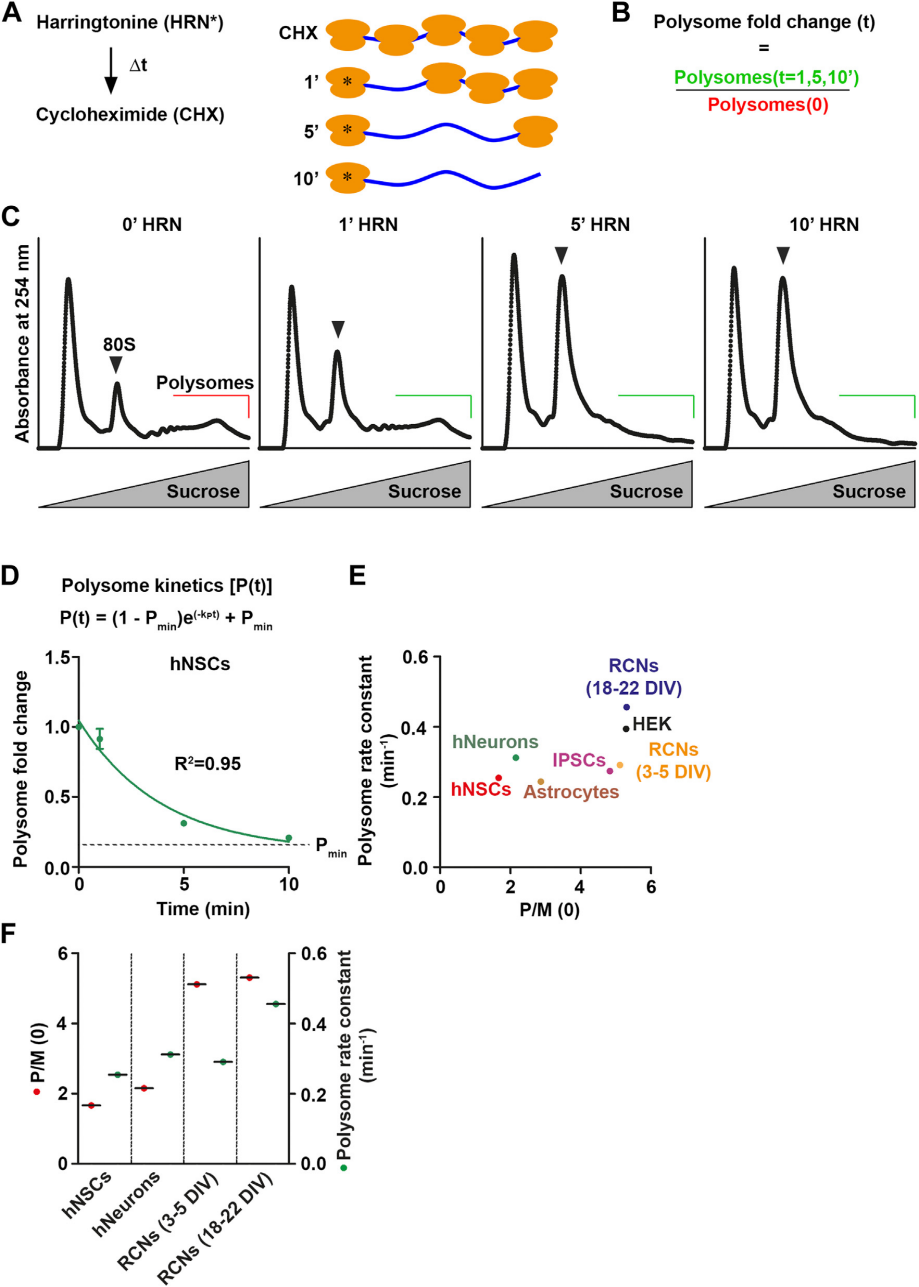
**“Ribosome runoff kinetics” to study ribosome speed.***A*, experimental scheme for polysome kinetics. *B*, polysome fold changes were determined by calculating the area under the polysome curve for each time point relative to the CHX control. *C*, representative polysome profiles of hNSCs used for kinetic studies. *D*, polysome kinetic curve of hNSCs used for calculating polysome rate constants k_P_. *E*, correlation between P/M(0) and polysome rate constants for the different cell types (n = 3 biological replicates, for mature RCNs n = 4 biological replicates). *F*, P/M(0) and polysome rate constants of hNSCs and immature RCNs during their *in vitro* development to human neurons and mature RCNs, respectively. Data are represented as mean ± SEM. CHX, cycloheximide; hNSCs, human neural stem cells; RCNs, rat cortical neurons.

**Figure S1:**
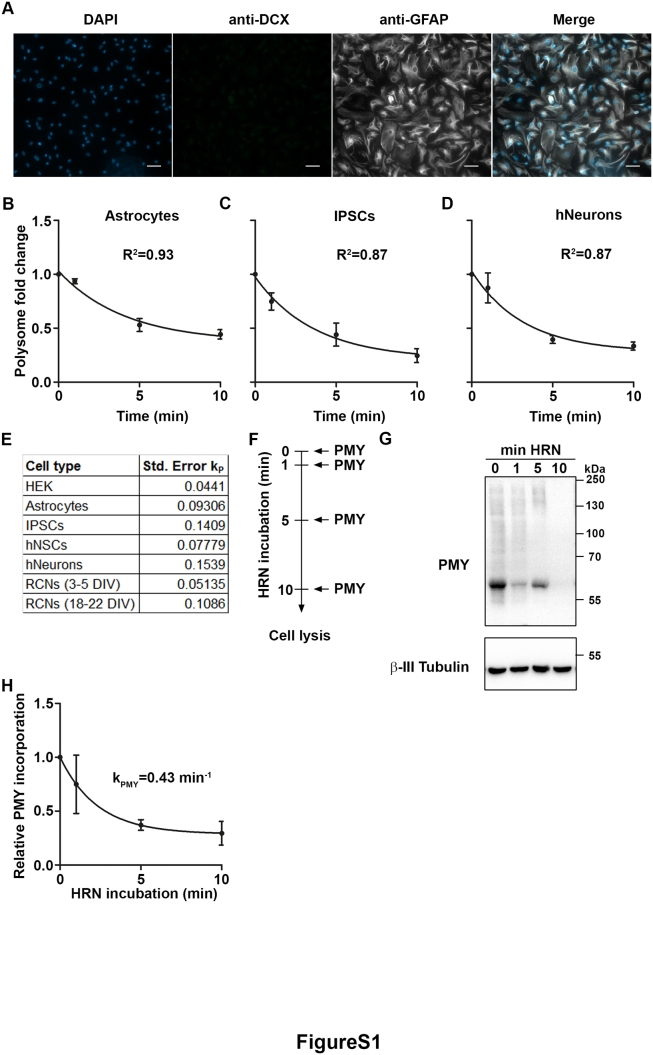
**Ribosome kinetics of different cell types.** Representative fluorescence microscopy images of cultured astrocytes stained against the neuronal marker doublecortin (DCX) and the glia cell marker glial fibrillary acidic protein (GFAP). DAPI was used to counterstain nuclei, Scale bar: 50 μm. *B*–*D*, Ribosome kinetics of astrocytes (*B*), IPSCs (*C*) and human neurons (*D*) (n = 3 biological replicates for each). *E*, standard errors for the polysome rate constants of the different cell types investigated. *F*, experimental scheme for puromycylation. (*G*/*H*) Representative immunoblot for PMY (*G*) using RCN lysate from runoff experiments and quantification of PMY incorporation (*H*). β-III Tubulin was used as loading control (n = 3 biological replicates).

To investigate global translation kinetics, we used the translation inhibitor harringtonine (HRN). HRN immobilizes initiating ribosomes at start codons by blocking the initial peptide bond formation and has been used to map translation start sites (43) as well as to study ribosome elongation speed (30, 43). For our analysis, we investigated human neural stem cells (hNSCs), both immature and mature rat cortical neurons (RCNs, 3–5 and 18–22 days *in vitro* (DIV), respectively). Furthermore, we utilized human induced pluripotent stem cells (IPSCs) and differentiated them into neurons (44). We also used cultured astrocytes, which were isolated from postnatal mouse brains (Fig. S1*A*). We also included kinetics data from HEK cells, which we previously published using the same experimental approach (45). To investigate speed of ribosomes engaged in polysomes, we incubated cells with pulses of HRN for 1, 5, and 10 min to induce ribosome runoff followed by cycloheximide (CHX) to stop all elongating ribosomes (Fig. 1*A*). Lysates were collected thereafter and subjected to polysome profiling. As nerve cells, in particular, contain heavy ribosome complexes that are dense and accumulate in the pellet during sucrose gradient centrifugation, we treated the lysates with deoxycholate (DOC) to dissolve them (refer Experimental procedures) (46). To monitor the decline in polysomes during HRN incubation time, we calculated the area under the polysome curves for each time point (Fig. 1, *B* and *C*). As expected, prolonged incubation with HRN resulted in increased monosome peaks and decreased polysome peaks, as shown for hNSCs (Fig. 1*C*). The fold changes of polysomes relative to CHX-treated controls (t = 0) were calculated during ribosome runoff (Fig. 1*B*), and plotted against the HRN incubation time (45). The exponential decay kinetics model (47) was used to fit the decline of polysomes (Fig. 1*D*). For all types of cells studied, a high coefficient of determination was observed indicating that polysome kinetics exhibit an exponential decay kinetics (Fig. S1, *B*–*D*). We approximated the ribosome speed by calculating the polysome rate constant k_P_ for each cell type (Fig. S1*E*). Moreover, we computed the polysome-to-monosome ratio for the CHX controls (P/M(0)). Notably, both P/M(0) and polysome rate constants differ between the cell types (Fig. 1*E*). As expected, HEK cells exhibit both high polysome rate constants and P/M(0) values. Interestingly, we found that mature RCNs (18–22 DIV) display an even higher k_P_ than HEK cells (Fig. 1*E*). We substantiated this rate constant using a complementary method employing nascent chain labeling with puromycin (PMY) during ribosome runoff (Fig. S1*F*). Upon immunoblotting with an anti-PMY specific antibody (48), we calculated the decrease in PMY incorporation and fitted the curve with an exponential decay kinetics. In accordance with our polysome profiling findings, the kinetics of PMY incorporation also exhibit a comparable rate constant (Fig. S1, *G* and *H*).

Additionally, our findings indicate an increase in the polysome rate constant during neuronal maturation, with the P/M(0) remaining mostly unaffected (compare immature with mature RCNs, Fig. 1, *E* and *F*). We observed a similar trend in the development of hNSC to human neurons (Fig. 1, *E* and *F*), albeit with a slight increase in the P/M(0). Collectively, these results highlight differences in ribosomal speeds across different cellular contexts. Moreover, neurons exhibit an acceleration of ribosomes during development, implying that translational speed plays a crucial role in nerve cell development (42).

### Neurons contain translationally inactive ribosomes

**Figure 2:**
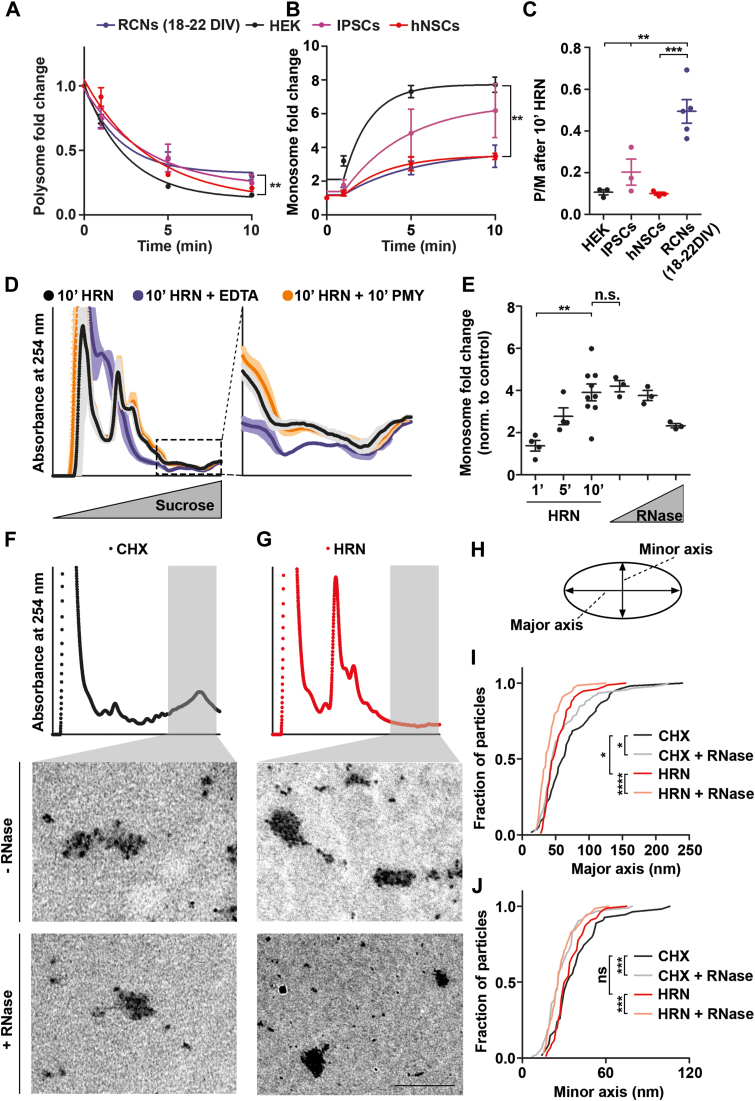
**Neurons contain inactive ribosomes.***A* and *B*, polysome (*A*) and monosome (*B*) kinetic curves for HEK, IPSCs, hNSCs, and mature RCNs. *C*, P/M after 10 min of HRN treatment for HEK cells, IPSCs, hNSCs, and mature RCNs (for all n = 3 biological replicates, for mature RCNs n = 5 biological replicates). *D*,polysome profiles of RCNs (18–22 DIV) treated either with HRN, HRN + EDTA or HRN + PMY. Shadows represent SEM (n = 3 biological replicates). *E*, monosome fold changes (relative to CHX or undigested control) upon treatment with HRN for different time points and upon treatment with different concentrations of RNase1, respectively (HRN treatment n > 3 biological replicates, RNAse treatment n = 3 biological replicates). *F* and *G*, representative transmission electron microscopy images of ribosomes isolated from polysomal fractions from CHX (*F*) and HRN (*G*) treated RCNs ± RNase (n = 3 biological replicates, the scale bar represents 500 nm). *H*, scheme of ribosome particles. Major and minor axis were measured. *I* and *J*, length of major (*I*) and minor (*J*) axis of CHX and HRN particles ± RNase, respectively. *p*-values were calculated using two-sided unpaired Student’s *t* test (*A* and *B*), One-way ANOVA with subsequent Tukey’s Multiple Comparison Test (*C* and *E*) or two-sided Mann-Whitney U test (*I* and *J*). ∗*p* < 0.05, ∗∗*p* < 0.01, ∗∗∗*p* < 0.001, ∗∗∗∗*p* < 0.0001. *Dots* represent independent replicates. Data are represented as mean ± SEM. n.s., not significant. CHX, cycloheximide; DIV, days *in vitro*; hNSCs, human neural stem cells; HRN, harringtonine; IPSCs, induced pluripotent stem cells; PMY, puromycin; RCNs, rat cortical neurons.

**Figure S2:**
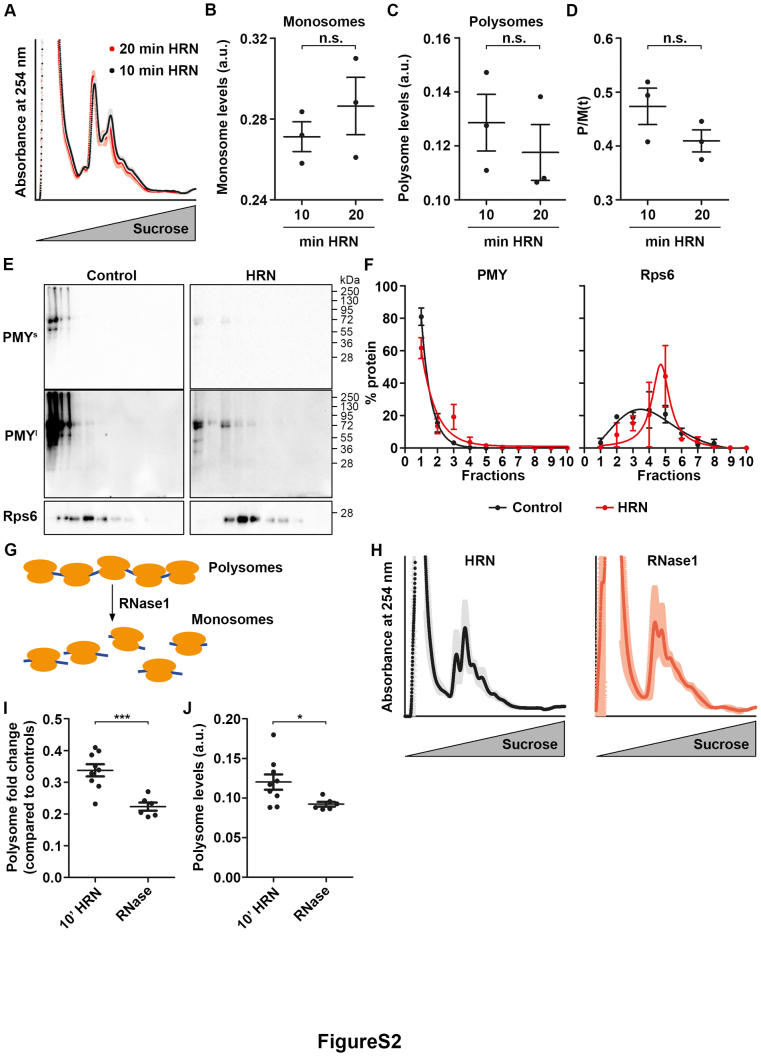
**Inactive ribosomes are RNase-sensitive.***A*, polysome profiles of RCNs (18–22 DIV) treated for 10 and 20 min with HRN, respectively. Shadows represent SEM of 3 biological replicates. *B*–*D*, Quantification of monosome (*B*) and polysome (*C*) levels as well as P/M ratio (*D*) after 10 and 20 min of HRN treatment, respectively (n = 3 biological replicates). *E* and *F*, Representative immunoblots for PMY-labelled proteins isolated from polysome profiles of control and HRN (10 min) treated mouse cortical neurons. Rps6 was used to detect the fate of ribosomes. s and l refer to short and long exposure, respectively. Quantifications are shown in (*F*, n = 2 biological replicates). *G*, cartoon for polysome digestion to produce ribosome protected fragments. *H*, representative polysome profiles of RCNs treated with HRN or RNase1. Shadows represent SEM (n > 3 biological replicates). *I* and *J*, Polysome fold changes relative to CHX control (*I*) and absolute polysome levels (*J*) after 10 of HRN treatment and RNase digestion, respectively (n > 6 biological replicates). *p*-values were calculated using two-sided unpaired Student’s *t*-test. ∗*p* < 0.05, ∗∗∗*p* < 0.001. Dots represent biological replicates. Data are represented as mean ± SEM. n.s., not significant.

As a next step, we wanted to understand the molecular mechanism of the accelerated ribosomal speed in neurons. We first compared the decline curves of polysomes in HEK cells, with hNSCs, IPSCs, and mature RCNs upon HRN treatment (Fig. 2*A*). Despite exhibiting similar P/M(0) values (Fig. 1*E*), mature RCNs exhibited a higher plateau in their decline curve than HEK cells resulting in a less pronounced decline in polysomes to the latter (Fig. 2*A*). This result indicates an incomplete runoff of neuronal polysomes. To exclude the possibility that a reduced decline in polysome abundance is due to detection limits, we also computed the increase in monosomes for the corresponding cell types during ribosome runoff (Fig. 2*B*). Our observations reveal that mature RCNs undergo less prominent fold changes as compared to HEK cells (Fig. 2*B*). Consequently, we found that RCNs had a significantly higher P/M after 10 min of ribosome runoff compared to other cells (Fig. 2*C*). These results imply that neurons exhibit incomplete runoff after 10 min of HRN. As some transcripts may have slower runoff (49), we conducted control experiments to investigate whether neurons have a greater proportion of slowly translating ribosomes than other cells. Initially, we examined whether a 10 min HRN incubation period leads to complete runoff. To this end, we incubated RCNs with HRN for 20 min and examined monosome and polysome levels as well as the P/M ratios. Crucially, we observed no statistically significant differences in either monosome and polysome levels or in P/M ratios between cells treated with HRN for 10 or 20 min (Fig. S2, *A*–*D*), indicating that 10 min of HRN treatment is sufficient for the vast majority of ribosomes to run off. As a next step, lysates treated with HRN were incubated with EDTA to disassemble ribosomes into ribosomal subunits. Interestingly, a reduction in RNA signal was observed in polysomal fractions upon EDTA treatment (Fig. 2*D*) implying that assembled ribosomes are present in these fractions. We further investigated whether these ribosomes were slowly translating by incubating cells with PMY upon HRN treatment. PMY is incorporated into nascent chains and as a result disassembles translationally active polysomes (50). Notably, PMY treatment did not lower the RNA signal within polysomal fractions of HRN-treated neurons (Fig. 2*D*). Consequently, these findings suggest that neurons contain translationally inactive polysomes. Given that stalled ribosomes are also insensitive to PMY (51), our next step was to test whether inactive ribosomes are stalled on mRNAs. Therefore, cortical neurons exposed to HRN treatment or control were incubated with PMY, and polysome profiling was performed. Puromycylated proteins were detected through immunoblotting using an anti-PMY specific antibody (48). As expected, we found that ribosome runoff significantly reduces the incorporation of PMY into nascent chains (Fig. S2*E*). Additionally, PMY positive nascent chains were not detected in heavy fractions of HRN polysome profile (Fig. S2, *E* and *F*), indicating that nascent chains are absent in translationally inactive ribosomes. Next, we used a complementary method to determine the levels of ribosomes bound to mRNAs. Polysomes were enzymatically digested to generate monosomes in the lysates (Fig. S2*G*). The resulting monosomes were generated from actively elongating (52) or stalled polysomes (53). The change in monosome levels relative to the undigested control was determined and compared to the change in monosome levels relative to the CHX control after HRN treatment. We observed no statistically significant difference between RNase1 treated and 10 min of HRN (Figs. 2*E* and S2*H*). Crucially, increasing RNase1 amounts did not lead to a higher but instead a lower monosome fold change, which suggests an over-digestion of ribosomes (Fig. 2*E*). Notably, despite similar increases in monosomes following RNase1 and HRN treatments, HRN-treated lysates exhibited higher relative and absolute polysome levels compared to those treated with RNase1 (Fig. S2, *I* and *J*). These findings collectively indicate the presence of ribosomal complexes in neurons that are inactive, sensitive to endonuclease digestion, do not produce ribosome footprints, and lack nascent chains.

**Figure 3:**
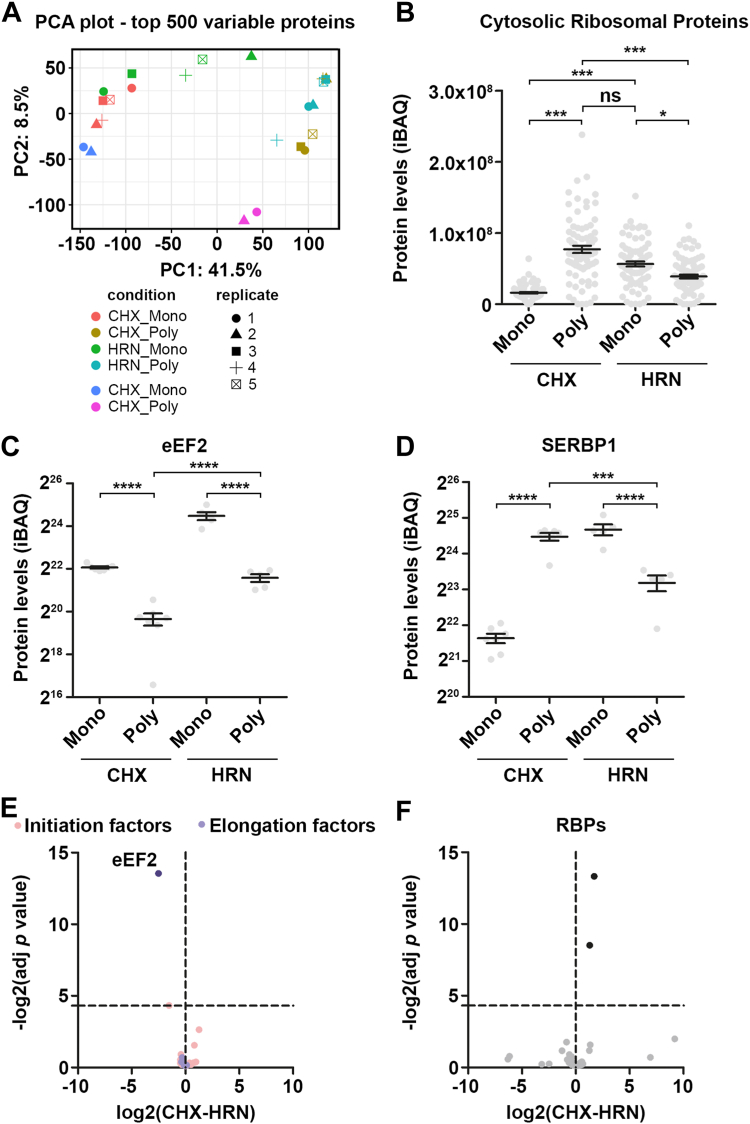
**The Ribo-proteome of elongating ribosomes.***A*, principal component analysis (PCA) of samples subjected to mass spectrometry. *B*, levels of cytosolic ribosomal proteins upon CHX and HRN treatment in monosomal and polysomal fractions. *C* and *D* protein levels of eEF2 (*C*) and SERBP1 (*D*) in monosome and polysome fractions upon CHX or HRN treatment. *E* and *F* fold changes in protein abundance of initiation and elongation factors (*E*) as well as RBPs (*F*) in polysomal fractions upon HRN treatment. *p*-values were calculated using Kruskal-Wallis test with subsequent Dunn’s Multiple comparison test (*B* and *D*), One-way ANOVA with subsequent Tukey’s Multiple Comparison Test (*C*) and see Experimental procedures. ∗*p* < 0.05, ∗∗∗*p* < 0.001, ∗∗∗∗*p* < 0.0001. *Dots* represent ribosomal proteins (*B*) or biological replicates (*C* and *D*). Data are represented as mean ± SEM. n.s., not significant. CHX, cycloheximide; eEF, eukaryotic elongation factor; HRN, harringtonine; RBPs, RNA-binding proteins; SERBP1, SERPINE1 mRNA-binding protein 1.

**Figure S3:**
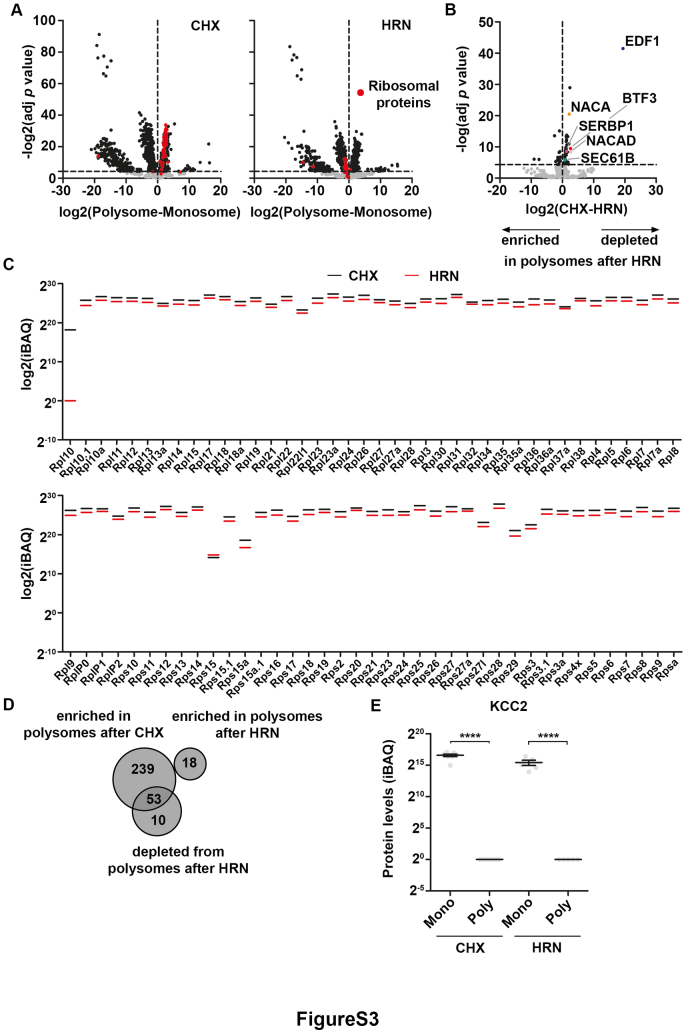
**Inactive ribosomes display almost complete ribosomal protein composition.***A*, volcano plots of proteins detected in mono- and polysomal fractions after CHX (*left*) and HRN (*right*) treatment, respectively. *B*, volcano plot for the fold change of proteins detected in polysomal fractions after CHX and HRN treatment. Ribosome associated proteins with known functions in protein synthesis are highlighted. *C*, ribosomal protein intensity in polysomal fractions of CHX and HRN treated RCNs. *D*, venn diagram showing the numbers proteins that are enriched in polysomal fractions upon CHX treatment and shift in response to HRN. *E* protein levels of KCC2 in monosome and polysome fractions after CHX and HRN, respectively. *p*-values were calculated using Kruskal-Wallis test with subsequent Dunn’s Multiple comparison test (*E*) and see Experimental procedures. ∗∗∗∗*p* < 0.0001. Dots represent individual proteins (*A*/*B*) or biological replicates (*E*). Data are represented as mean ± SEM.

To visualize inactive ribosomes, we conducted transmission electron microscopy (TEM). To this end, we isolated ribosomes from polysomal fractions of CHX and HRN-treated RCNs (18–22 DIV, Fig. 2, *F* and *G*). Heavy fractions from CHX-treated cells displayed typical polysomal electron densities (Fig. 2*F*). In contrast, we observed densely packed ribosomes condensed in granule-like structures upon runoff of active ribosomes (Fig. 2*G*). To analyze our TEM data further, we quantified the major and minor axis of electron dense particles (Fig. 2*H*). Interestingly, we found that HRN particles have a reduced major axis but not a reduced minor axis compared to CHX ribosome particles (Fig. 2, *I* and *J*), suggesting these granules have a different geometry. Furthermore, both particles were susceptible to RNase treatment (Fig. 2, *F*, *G*, *I* and *J*). To further support the presence of fully assembled ribosomes within these ribosomal complexes, we analyzed the protein composition of polysomal fractions from cells treated with CHX or HRN. We isolated proteins from monosome and polysome fractions of CHX or HRN-treated RCNs (18–22 DIV) and conducted label-free mass spectrometry analysis (Dataset S1). Proteins bound to or associated with elongating ribosomes undergo a shift from polysomal, heavy fractions to the lighter monosome fraction. Principal component analysis revealed that samples were separated by their sedimentation through the sucrose gradient but not by the drug treatment, indicating that only a subfraction of proteins was affected by ribosome runoff (Fig. 3*A*). Indeed, we observed that ribosomal proteins were enriched in CHX polysomes (average log2FC = 2.2, Fig. S3*A*) and shifted toward monosome fractions in HRN profiles (Fig. 3*B*), as expected. Importantly, although there was a shift of cytosolic ribosomal proteins toward lighter fractions upon runoff, significant levels of almost all of these proteins still remained in the polysomal fractions (Figs. 3*B* and S3*C*). These findings corroborate our electron microscopy data (Fig. 2, *F*–*J*) and indicate that inactive, fully assembled ribosomes accumulate in densely packed granule-like structures in neurons.

### eEF2 is associated with inactive ribosomes

We analyzed proteins that were enriched in heavy, polysomal fractions in CHX over HRN cells (Dataset S2). In total, we observed 292 proteins with a higher abundance in polysome compared to monosome fractions under CHX treated conditions. Of these, 35% were ribosomal or ribosome bound proteins, 16% were RBPs, and 12% were splicing factors. Fifty-three out of 292 proteins shifted from polysomes to monosomes upon HRN induced runoff, with most being ribosomal proteins but also including RBPs like SERPINE1 mRNA-binding protein 1. Additionally, known ribosome associated proteins like BTF4, NACA, NACAD, and EDF1 showed enhanced abundance in CHX in contrast to HRN polysomal fractions (Fig. S3*B*). For gaining insights on why inactive ribosomes are retained in neurons, we determined proteins that remain associated with polysomal fractions after 10 min of HRN. We noticed 18 proteins that displayed elevated levels in polysomes when compared to CHX (Fig. S3*D*). One of these proteins is eEF2 (Fig. 3*C*), which demonstrated an increase in monosomal and polysomal fractions upon HRN induced runoff. This effect was distinct from the sedimentation pattern of proteins that do not associate with elongating ribosomes like the chloride-ion transporter KCC2 (Fig. S3*E*) or ribosome bound proteins like SERPINE1 mRNA-binding protein 1, which showed reduced polysomal levels upon HRN treatment (Fig. 3*D*). To determine whether the increased levels of eEF2 found in polysomal fractions indicate a general response of elongation factors (EFs) or initiation factors (IFs) to ribosome runoff, we analyzed all EFs and IFs in our dataset, but none showed a comparable effect (Fig. 3*E*). As translation activity crucially contributes to RNA granule formation (18), we also analyzed RBP sedimentation (Fig. 3*F*). We discovered that none of the RBPs detected displayed a bias toward polysomal fractions after ribosome runoff, suggesting that RNA granule formation does not drive eEF2 association with these large ribosomal complexes.

### eEF2 levels influence the amount of translationally inactive ribosomes

**Figure 4:**
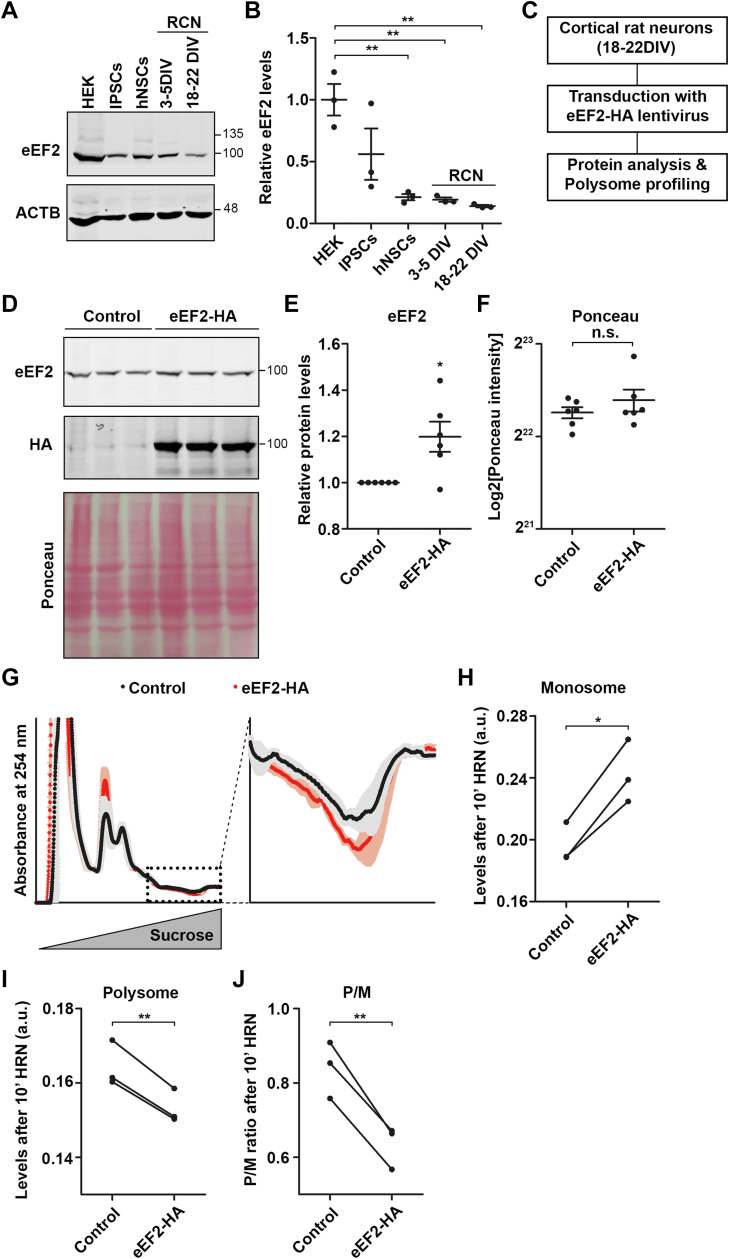
**eEF2 levels regulate numbers of inactive ribosomes.***A* and *B*, representative immunoblots for eEF2 from lysates of HEK cells, IPSCs, hNSCs, and immature and mature RCNs (*A*) and quantification (*B*). ACTB was used as loading control (n = 3 biological replicates). *C*, experimental scheme for eEF2 overexpression in RCNs. *D*–*F*, representative Western blot for eEF2 and HA from lysates of control and eEF2-HA transduced RCNs (*D*) as well as eEF2 quantification (*E*). Ponceau staining shows protein loading (*D* and *F*). Same number of cells was used for Western blotting (n = 6 biological replicates). *G*, polysome profiles of control and eEF2-HA transduced RCNs after 10 min of HRN treatment. *Shadows* represent the SEM (n = 3 biological replicates). *H*–*J*, quantification of monosomal (*H*) and polysomal levels (*I*) as well as P/M (*J*) from polysome profiles of eEF2-HA transduced and control RCNs after 10 min of HRN incubation. *p*-values were calculated using One-way ANOVA with subsequent Tukey’s multiple comparison test (*B*), one-sample *t* test (*E*), two-sided unpaired *t* test (*F*) and two-sided paired Student’s *t* test (*H*–*J*). ∗*p* < 0.05, ∗∗*p* < 0.01. *Dots* represent biological replicates. Data are represented as mean ± SEM. n.s., not significant. ACTB, beta actin; eEF, eukaryotic elongation factor; HA, hemagglutinin; HEK, human embryonic kidney; hNSCs, human neural stem cells; HRN, harringtonine; IPSCs, induced pluripotent stem cells; RCNs, rat cortical neurons.

**Figure S4:**
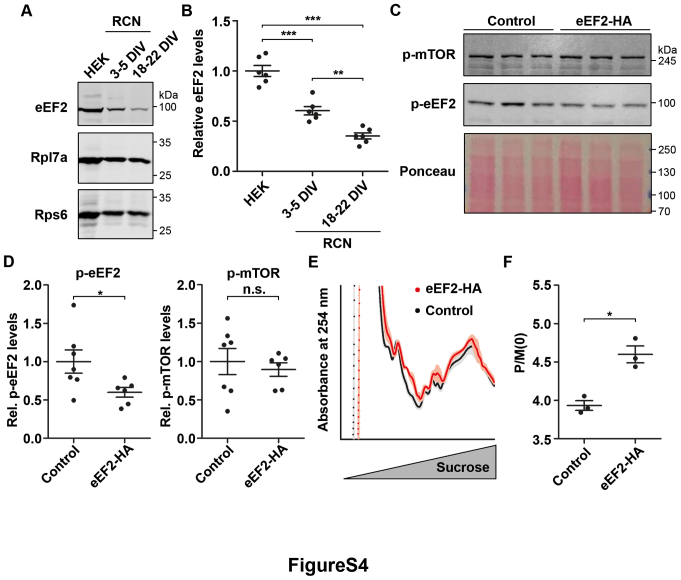
**Increasing eEF2 levels enhance translation.***A* and *B*, Representative immunoblots for eEF2, Rpl7a and Rps6 (*A*) using lysates from HEK, immature and mature RCNs as well as their quantifications (*B*). Ribosomal proteins were used as loading controls (n = 6 biological replicates). *C* and *D*, Representative immunoblots against p-eEF2 and p-mTOR (*C*) using lysates from control and eEF2-HA transduced RCNs (18–22 DIV) as well as quantification (*D*). Ponceau served as loading control (n = 7 biological replicates for control, n = 6 biological replicates for eEF2-HA). *E*, polysome profiles (CHX treated) of control and eEF2-HA transduced RCNs (18–22 DIV). Shadows represent SEM of 3 biological replicates. *F*, steady-state P/M(0) upon eEF2-HA overexpression in RCNs (18–22 DIV, n = 3 biological replicates). *p*-values were calculated using One-way ANOVA with subsequent Tukey’s Multiple comparison test (*B*) and two-sided paired Student’s *t*-test (*D* and *F*). ∗*p* < 0.05, ∗∗*p* < 0.01, ∗∗∗*p* < 0.001. Dots represent biological replicates. Data are represented as mean ± SEM. n.s., not significant.

Our results prompted us to gain further insight into the impact of eEF2 on translation. First, we analyzed the steady-state levels of eEF2 in various cells, which exhibited lower inactive ribosome levels compared to RCNs (Fig. 2, *A*–*C*). We found that the expression of eEF2 was comparatively lower in RCNs than in HEK cells, as confirmed by the use of either beta actin (ACTB) (Fig. 4, *A* and *B*) or ribosomal proteins as loading control (Fig. S4, *A* and *B*). Importantly, this finding is consistent with polysome sequencing data from human neurons derived from human embryonic stem cells, which showed decreased translation of *eEF2* mRNA during neuronal differentiation (54). Our ribosome proteomic data and the eEF2 levels led us to speculate that eEF2 levels may be limiting in neurons. Consequently, during runoff, eEF2 is released from ribosomes that underwent active translation to interact with translationally inactive ribosomes. Therefore, we sought to investigate whether elevated eEF2 levels could counteract ribosome inactivation and enhance active translation. To achieve this, we ectopically expressed hemagglutinin (HA)-tagged eEF2 in RCNs (18–22 DIV) using lentiviruses (Fig. 4*C*). While we were able to effectively express eEF2-HA in RCNs, we only observed a modest, albeit statistically significant, increase of total eEF2 protein (Fig. 4, *D*–*F*). In contrast, the inactive, phosphorylated form of eEF2 exhibited a more pronounced decrease (Fig. S4, *C* and *D*). As a result, we observed that the ectopic expression of eEF2-HA augmented the steady-state P/M ratio (Fig. S4, *E* and *F*), indicating increased translational activity. This effect was not dependent on phospho(p)-mTOR levels which remained unaltered by the ectopic expression of eEF2-HA (Fig. S4, *C* and *D*). We conducted further ribosome runoff experiments using neurons that ectopically expressed eEF2-HA. Our findings indicate that the upregulation leads to a reduction in inactive ribosomes, which is supported by the decrease in RNA signal in polysomal fractions after 10 min of HRN treatment (Fig. 4, *G* and *I*). Further to the decrease in inactive ribosomes, we observed an increase in monosomes (Fig. 4, *G* and *H*) and subsequently a decrease in the P/M after runoff (Fig. 4*J*). These observations imply that restraining eEF2 activity promotes ribosome inactivation. Therefore, the availability of active neuronal eEF2 is paramount for balancing active and inactive ribosomes and, consequently, ribosome elongation velocity.

### Neuronal excitation remodels the dynamics of translation

**Figure 5:**
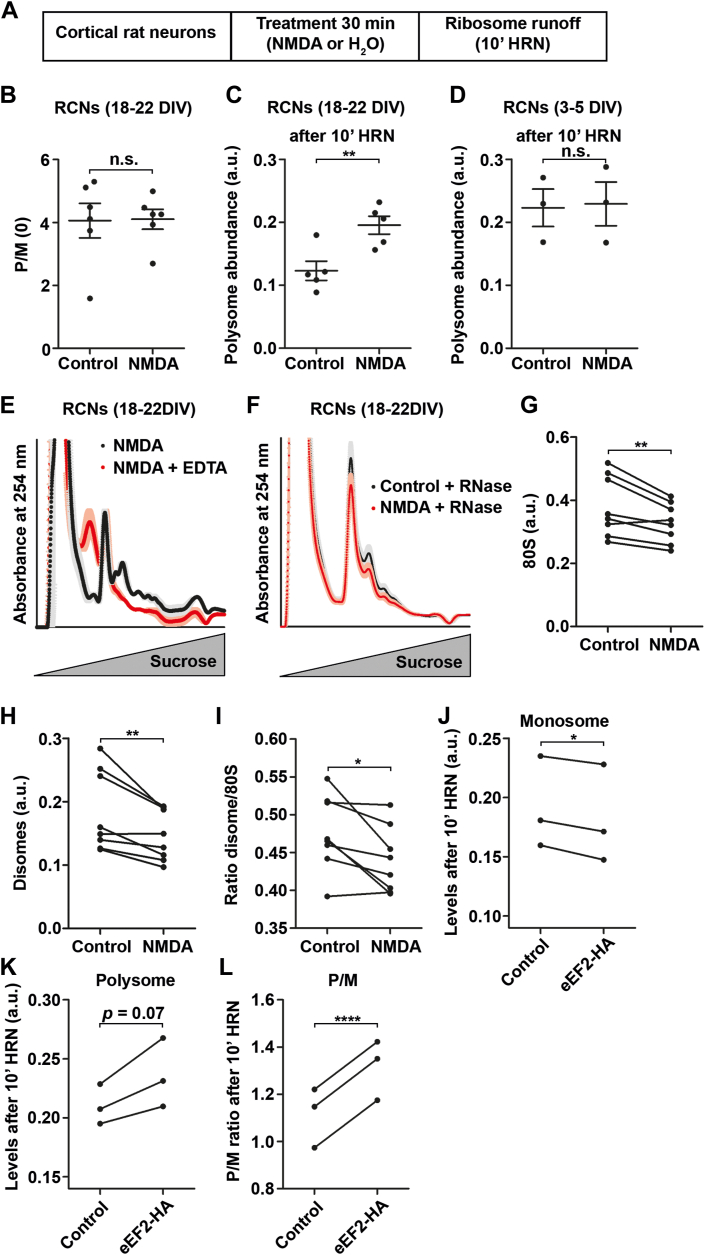
**Neuronal stimulation inactivates ribosomes**. *A*, experimental scheme for neuronal stimulation with subsequent ribosome runoff. *B*, steady-state translation activity (P/M(0)) of NMDA stimulated neurons compared to controls (n = 6 biological replicates). *C* and *D*, absolute polysome levels upon ribosome runoff of control and NMDA treated mature (18–22 DIV, *C*) and immature (3–5 DIV, *D*) RCNs (n = 3 biological replicates for immature RCNs, n = 5 biological replicates for mature RCNs). *E*, polysome profiles of NMDA incubated RCNs after 10 min of runoff either mock or EDTA treated. *Shadows* represent SEM (n = 3 biological replicates). *F*, polysome profiles of NMDA treated and control neurons after RNase1 treatment. *Shadows* represent SEM (n = 8 biological replicates). *G*–*I*, quantification of 80S (*G*) and disome (*H*) as well as their ratio (*I*) from polysome profiles depicted in (*F*) (n = 8 biological replicates). *J*–*L*, quantification of monosomal (*J*) and polysomal levels (*K*) as well as P/M (*L*) from polysome profiles of eEF2-HA transduced and control RCNs treated with NMDA after 10 min of ribosome runoff (n = 3 biological replicates). *p*-values were calculated using two-sided unpaired Student’s *t* test (*B*, *C* and *D*) or two-sided paired Student’s *t* test (*G*–*L*). ∗*p* < 0.05, ∗∗*p* < 0.01, ∗∗∗∗*p* < 0.0001. *Dots* represent biological replicates. Data are represented as mean ± SEM. n.s., not significant. DIV, days *in vitro*; eEF, eukaryotic elongation factor; HA, hemagglutinin; NMDA, N-methyl-D-aspartate; RCNs, rat cortical neurons.

**Figure S5:**
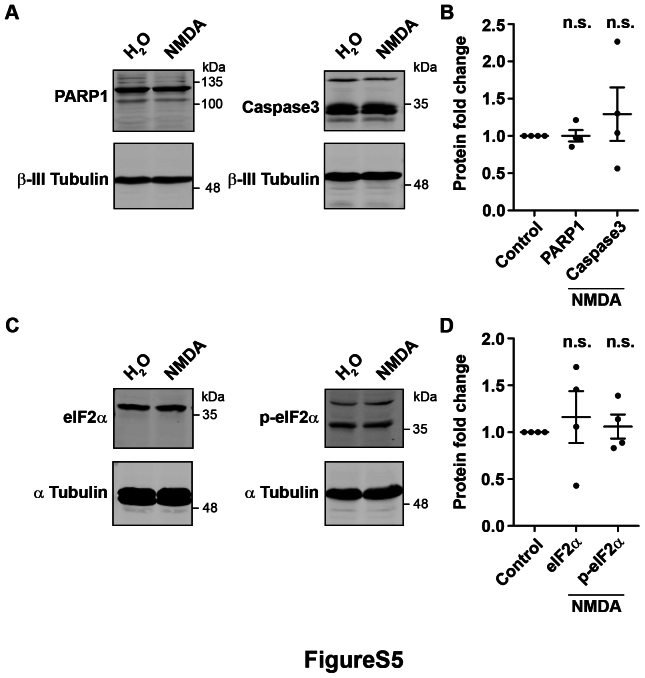
**NMDA treatment does not induce apoptosis.***A* and *C*, representative immunoblots for apoptotic markers PARP1 and Caspase3 (*A*) as well as the intrinsic stress response regulator (p)-eIF2a (*C*) from lysates of RCNs treated either with water or NMDA. Quantifications are shown in (*B* and *D*). β-III Tubulin and α Tubulin were used as loading controls, respectively (n = 4 biological replicates). *p*-values were calculated using one-sample *t* test. Data are represented as mean ± SEM. n.s., not significant.

Our findings indicate that neurons inactivate a fraction of their ribosomes to compensate for lower levels of eEF2. As the next step, our goal was to comprehend the neuronal response to stimuli that remodel their translatome. Synaptic activity is known to remodel the translatome and proteome globally (55, 56, 57), which has the potential to cause excitotoxicity (58). This involves translational upregulation of certain transcripts while others are silenced. To investigate the impact on translation, we excited RCNs (18–22 DIV) with N-methyl-D-aspartate (NMDA). To elicit a strong effect, we incubated RCNs with 100 μM NMDA for 30 min (Fig. 5*A*). Importantly, neurons survive these conditions without showing significant increase in apoptosis up to 12 h after treatment (58). To test that NMDA exposure for 30 min did not induce apoptosis under our conditions, we measured levels of the apoptotic markers PARP1 and caspase3. We determined that RCNs treated with NMDA for 30 min did not exhibit any effect on both markers (Fig. S5, *A* and *B*). Furthermore, we observed no changes in the levels of total and phosphorylated eIF2α, which is a regulator of the integrated stress response (59) (Fig. S5, *C* and *D*). Additionally, we also observed no differences in the P/M(0) values (Fig. 5*B*). Subsequently, we investigated the effect of neuronal excitation on the levels of translationally inactive ribosomes. To achieve this, we treated RCNs with NMDA and performed ribosome runoff experiments (Fig. 5*A*). We computed the polysome levels after ribosome runoff (Fig. 5*C*). We observed that RCNs treated with NMDA displayed higher levels of RNA in polysomal fractions, indicating increased levels of inactive ribosomes. To determine whether this effect is reliant on mature synapses, we treated young, immature RCNs (3–5 DIV) with NMDA and observed no impact on RNA levels in polysome fractions (Fig. 5*D*). Furthermore, we discovered that the increase in polysomal RNA induced by NMDA is mediated by ribosomes, as treatment of lysates with EDTA decreased the signal (Fig. 5*E*). To investigate whether this increase in RNA signal originates from slowly translating or inactive ribosomes, we treated lysates from control and NMDA-treated RCNs with RNase1. Alongside the rise in polysomal RNA signal, a reduction of 80S monosomes was observed in cells treated with NMDA (Fig. 5, *F* and *G*). Additionally, disomes decreased after NMDA treatment (Fig. 5*H*) and were more affected than monosomes (Fig. 5*I*). These findings imply that NMDA treatment diminishes the number of translationally active ribosomes while increasing the levels of inactive ribosomes. Our data led us to hypothesize that neuronal stimulation results in less demand for active ribosomes. Therefore, redundant ribosomes are stored as inactive ribosomes within ribosomal granules. One implication of this hypothesis would be that the rise in the number of active ribosomes before NMDA treatment would lead to increased accumulation of inactive ribosomes. In order to verify this, we utilized our eEF2-HA lentiviruses. We transduced RCNs (18–22 DIV) with eEF2-HA or control lentiviruses. After 4 days, the RCNs were subjected to NMDA treatment for 30 min, and ribosome runoff was carried out for 10 min. We observed that increasing levels and activity of eEF2 results in elevated levels of inactive ribosomes in NMDA-treated cells as evidenced by decreased monosome levels (Fig. 5*J*), increased polysome levels and P/M ratios (Fig. 5, *K* and *L*) after ribosome runoff.

**Figure 6:**
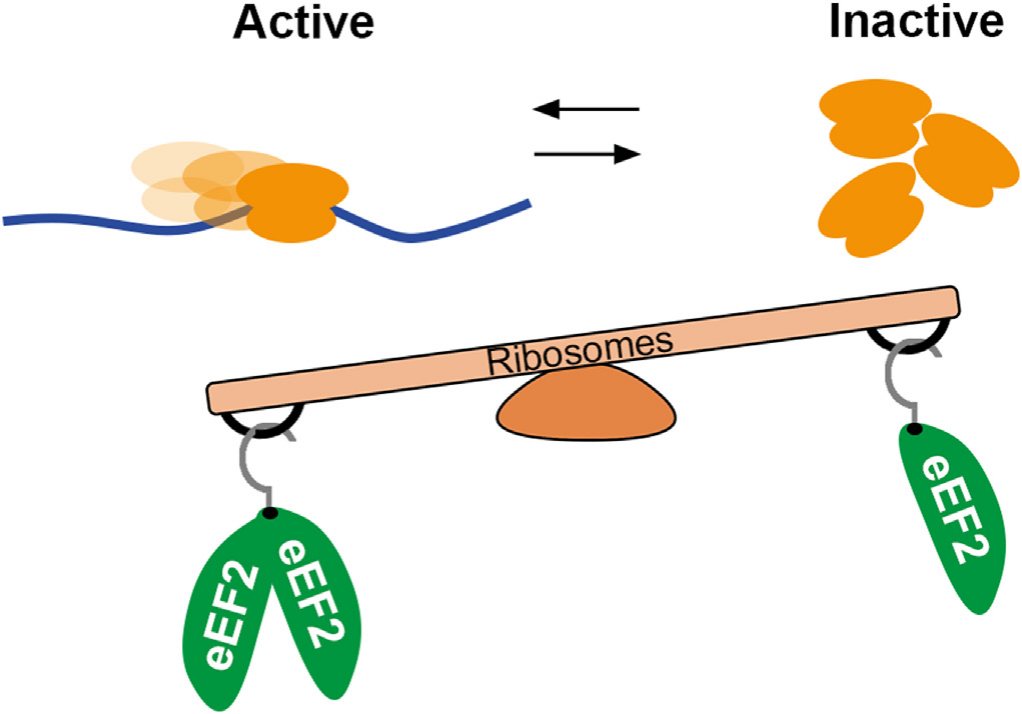
**eEF2 balancesribosome activity.** Our findings indicate that eEF2 plays a role in regulating the levels of translationally inactive ribosomes. Cells expressing high levels of eEF2 preferentially activate ribosomes rather than promoting their inactivation. By contrast, neurons exhibit lower levels of eEF2. As a result, eEF2 is limited in these cells, leading to ribosome inactivation and accumulation of inactive ribosome granules in the cytosol. eEF, eukaryotic elongation factor.

Together, these findings indicate that neurons contain ribosomes that are translationally inactive. The inactivation of ribosomes is governed by the levels of eEF2. Elevated eEF2 activity promotes ribosome activation and augments the levels of translationally active ribosomes (Fig. 6). Processes like neuronal excitation, which decrease the demand for active ribosomes, result in the accumulation of redundant, inactive ribosomes. Hence, higher levels of active ribosomes prior to NMDA stimulation lead to enhanced ribosome silencing.

## Discussion

### Ribosome speed varies across cell types

The human body comprises a range of cell types, each with a distinct function. To perform these functions, cells adjust their proteome (60) and thereby translate one subset of mRNAs over others (61, 62, 63, 64, 65). Although recent progress in single cell RNA sequencing (66, 67, 68) and ribosome profiling (69) has demonstrated the diversity of gene expression in different cell types, differences in translation rates are still poorly understood. This study aimed to explore translation dynamics in different cellular contexts. Our findings unveil different trends in ribosome elongation speed among various cell types. Importantly, distinct cell types and tissues exhibit different levels of transcriptome complexity (67, 70), which may influence translational activity and, subsequently, ribosomal speed. One aspect that can potentially influence ribosome speed is the length of the coding sequence (CDS) and the 3′-UTR, as has been shown for HEK cells (71). Additionally, tRNA concentration has a direct impact on ribosome speed (4). It is noteworthy that CDS and 3′-UTR length as well as tRNA levels, vary across tissues and cells (72, 73, 74), indicating a likely effect on translation velocity. Furthermore, it is possible that ribosome stalling (75, 76) and elongating monosomes (77, 78) are specific to certain cell types. Additionally, slowly translating ribosomes exist that show runoff even after 10 min of HRN (49). As we did not observe further ribosome runoff by increasing the HRN incubation time, we concluded that their abundance is too low to be captured by our polysome profiling experiments in contrast to the ribosome profiling approach used by Shah *et al.* (49) to identify slowly translating ribosomes.

Notably, variations in ribosome speed could be caused by varying protein degradation rates in different cell types (79), particularly between glia cells and neurons (80). Furthermore, the abundance of crucial *trans*-acting regulators like RBPs also differ between cells (81) and can regulate both translation activity and speed (3). One prominent example is the fragile X mental retardation protein, which binds to ribosomes and influences their translocation during protein synthesis (11, 49). We did not detect fragile X mental retardation protein in our ribosome proteomics dataset, presumably due to its sensitivity to DOC (82). In future experiments, however, its effect on ribosome inactivation should be considered.

Interestingly, we also found a switch from slowly to quickly translating polysomes during the development of RCNs. A similar effect was also observed in human neurons derived from neural stem cells. It is known that during neuronal development cells remodel their translatome (54). However, the stage of translation at which this occurs is largely unknown (83). Our data suggest that developing neurons decelerate ribosomes. As certain protein complexes are assembled cotranslationally (32), it is tempting to speculate that developing neurons require additional elongation time to facilitate correct protein localization and complex formation for proper cell fate commitment. This may not be crucial for fully developed, mature neurons, as local transcriptomes and translatomes are established (3). Consequently, these cells accelerate their ribosomes. The transition from slowly to quickly elongating ribosomes could be of significant clinical relevance. Complex neuropsychiatric and neurological diseases such as autism are characterized by a multitude of nonsynonymous mutations. Nonetheless, patients also exhibit synonymous, so-called, silent mutations (84). Silent mutations within codons can render ribosome speed if the levels of the cognate tRNAs binding to mutant or WT codons are different (85). Such mutations can alter cotranslational folding trajectories leading to protein degradation and/or aggregation (33, 34). It is therefore plausible that silent mutations may also play a role in the development of neurological and neuropsychiatric diseases. This aspect is of particular interest for neurons as they adjust their elongation rates during development. With the availability of new sequencing approaches for tRNAs (74), this aspect of translational dysregulation in neuropsychiatric diseases has become experimentally accessible (86).

### eEF2’s dual role in translation

During elongation, eEF2 stabilizes the ribosome hybrid state and induces conformational changes necessary to move one codon further (28). Therefore, eEF2 availability is essential for sufficient translation (45). Neurons express eEF2 at low levels. From our ribosome proteomics, we concluded that eEF2 is released from actively elongating ribosomes during runoff and can associate with inactive ribosomal granules. Aligned with this notion is our finding that as eEF2 levels rise, the amount of inactive ribosomes decreases. Based on these findings, we suggest that eEF2 levels serve as a regulatory hub for maintaining balanced ribosome activity. Elevated eEF2 levels permit adequate, active translation. Conversely, in conditions, such as in neurons, where eEF2 molecules are limited, some ribosomes might not receive adequate amounts of eEF2, which, in turn, could lead to ribosome inactivation. In addition to levels of eEF2, we found that neuronal excitation also regulates the inactivation of ribosomes. This effect of NMDA appears to be at first counter intuitive, as synaptic stimulation has been shown to increase protein synthesis (87, 88). However, synaptic stimulation affects only some proteins and eventually mRNAs (55), therefore it is possible that NMDA treatment alters the translatome to promote the translation of specific mRNAs, making some ribosomes unnecessary. Redundant ribosomes are stored in ribosomal granules. This effect is partially reliant on eEF2 levels. Ribosome inactivation increased when eEF2 was increased before NMDA stimulation. It is possible that cells that overexpress eEF2 possess a larger number of active ribosomes that become redundant upon NMDA treatment. Hence, these findings emphasize the crucial function of eEF2 in balancing ribosome activity. The importance of eEF2 in regulating ribosome activity has been highlighted by recent studies demonstrating that mutations in eEF2 in patients suffering from neurodevelopmental or neurodegenerative diseases impair translation fidelity (89, 90, 91). It is noteworthy that eEF2 availability affects the amount of inactive ribosomes, but it does not function as the sole regulator mechanism. For instance, hNSCs show relatively modest eEF2 levels (Fig. 4*A*), but fewer dormant ribosomes are present (Fig. 2*C*). Thus, it is plausible that additional factors, such as codon usage, RNA secondary structures, and tRNA abundance could also have an impact (6).

### Neurons contain translationally inactive ribosomes

The data presented in this study show that neurons contain translationally inactive ribosomes that are regulated by synaptic activity. Although our data do not allow us to distinguish between stalled and dormant, vacant ribosomes, our data suggest a novel type of dormant ribosomes that assemble into high-density granules and are devoid of mRNAs and nascent chains. In our proteomics approach, we did not detect ribosome quality control factors that were identified in a previous study using translational stress (92). Consequently, ribosome inactivation also occurs under physiological conditions in neurons as ribosome stalling (75). Nonetheless, the main query still stands: what is the purpose of these inactive ribosomes? Protein synthesis is a resource-intensive process that requires many factors and regulators (93). Therefore, neurons store certain molecules including mRNAs and translation regulators in ribonucleoprotein particles to enable protein synthesis remotely and on demand (3). Consequently, the existence of ribosome granules that store ribosomes in addition to ribonucleoprotein particles is conceivable. Nerve cells produce some of the proteins in response to synaptic activity (87, 88). A continuous turnover of these granules is therefore necessary to ensure adequate protein synthesis. Ribosome granules may play a crucial role in localized translation within neurons. Such translation is believed to be vital in modifying the synaptic proteome in response to synaptic stimulation. Therefore, it may be of utmost importance to maintain a dynamic equilibrium between ribosome activation and inactivation to ensure sufficient protein synthesis. As a portion of localized mRNAs is translated by a single ribosome (77, 78), it is conceivable that silencing the ribosome directly impacts the rate of protein synthesis of these transcripts. Future experiments should aim to investigate the effect(s) of ribosome inactivation on the local translatome and its consequential influences on synaptic plasticity and cognitive function. Clarifying these relationships will help to better understand the underlying mechanisms of memory formation.

### Limitations of this study

While polysome profiling is the preferred method to investigate global translation activity of cells and tissues, it has limitations in quantifying ribosome speed due to the qualitative nature of the method and the lack of transcript-specific information. Additionally, our approach does not enable us to study elongating monosomes, which translate a significant fraction of the entire translatome (77, 78). This might be important for cells that possess a greater number elongating monosomes, such as neurons (77, 78).

## Experimental procedures

### Cell culture

#### Primary cortical neurons

RCNs were isolated from E17.5 embryonic rats as previously described (94). In brief, cortices were manually dissected and trypsinized. Upon dissociation of tissue, cells were filtered using 100 μm, 70 μm, and 40 μm pore size filters. Two million cells were plated on poly-l-lysine–coated 6 cm dishes (95). All animals were used according to the German Welfare for Experimental Animals (LMU Munich, *Regierung von Oberbayern*).

Cortical mouse neurons were isolated from E14.5 C56BL/6J mouse embryos. Cortices were incubated in Dulbecco's modified Eagle's medium (DMEM)/F12, 0.0125 M glucose, 1% P/S, 1 mM EDTA for 5 min and subsequently trypsinized with 0.25% trypsin at 37 °C for 10 min. Reaction was stopped in DMEM/F12, 0.0125 M glucose, 1% P/S, 20% fetal bovine serum. Samples were dissociated in DMEM/F12, 0.0125 M glucose, 1% P/S, 10% fetal bovine serum, 4 KU/ml DNAseI using fire-polished glass pipette and then filtered through a 70 μm cell strainer nylon filter. Cells were counted, centrifuged for 5 min at 950 rpm in ALC PK120R centrifuge at room temperature and resuspended in complete Neurobasal medium (Neurobasal, 2% B27 supplement, 18 mM Hepes, 1% P/S, 0.5 mM L-glutamine). Neurons were seeded on poly-D-lysine and laminin coated plates. Half medium was exchanged every 48 h. Mice were housed within the animal care facilities at the University of Trento under appropriate conditions. Animal breeding and procedures were conducted under appropriate project and personal license granted by the ethical committee of the University of Trento and were approved by the Italian Ministry of Health (D. Lgs no. 2014/26, implementation of the 2010/63/UE).

#### hIPSCs, hNSCs, and hNeurons

hIPSCs were differentiated in hNSCs and hNeurons as previously described (44). In brief, IPSCs were cultured on Geltrex (Thermo Fisher Scientific) or Matrigel (Corning Life Sciences) coated dishes and cultured in mTESR medium. Medium was changed daily. For neuronal cell fate commitment, 2 to 3 million IPSCs were plated on Geltrex coated dishes. Cell differentiation was performed using a dual SMAD inhibition protocol (96). On day 10, cells were split and cultured till day 15 in N3 medium without SMAD inhibitors to generate hNSCs. To generate human neurons, cells were split on day 15 and day 27 and cultured in N3 medium without SMAD inhibitors until day 35/36.

#### HEK cells

HEK cells (American Type Culture Collection (ATCC) CRL-3216, authentication by ATCC) were cultured in DMEM + fetal calf serum medium at low passage number. Three million cells were used for experiments.

#### Astrocytes

Astrocytes were cultured as previously described (97). In brief, astrocytes were obtained from the gray matter of postnatal day 6 mouse cerebral cortex upon removing white Matter, ventricular regions and meninges. Cells were cultured in DMEM + F12 (Gibco) supplemented with epidermal growth factor and basic fibroblast growth factor. After 9 days, 800.000 cells were seeded on poly-D-lysine-coated dishes and cultured in DMEM + F12 supplemented with epidermal growth factor and basic fibroblast growth factor for 3 days.

### Pharmacological treatment

To stimulate cortical neurons, cells were treated with 100 μM NMDA (Biomol) or water as control for 30 min.

### Polysome kinetics

For polysome kinetics, cells were incubated with 2 μg/ml HRN (Biomol) for 1, 5, and 10 min. Ribosome runoff was stopped by adding 100 μg/ml CHX (Roth) after the indicated time points. As control, cells were treated only with CHX. Cells were washed three times with Hanks' Balanced Salt Solution supplemented with 100 μg/ml CHX and subjected to polysome profiling.

### PMY kinetics

Puromycylation kinetic experiments were performed as previously described (45). In brief, RCNs (18–22 DIV) were incubated for 1, 5, and 10 min with HRN, respectively. Nascent chains were labeled with 25 μM PMY for 10 min. For the “0” time point, cells were incubated with 25 μM PMY for 10 min. Upon incubation, cells were lysed in hot SDS buffer and PMY labeled proteins detected using immunoblotting.

### Polysome profiling

Polysome profiling was done as previously described (95, 98). In brief, cells were lysed in polysome lysis buffer (150 mM NaCl, 5 mM MgCl_2_, 10 mM Tris-HCl pH 7.4, 1 vol% NP-40, 1% (w/v) sodium deoxycholate supplemented with 100 μg/ml CHX and 2 mM dithiothreitol, DTT) on ice. Lysates were precleaned by spinning at 13,000*g* for 5 min at 4 °C and loaded onto a sucrose gradient (18% (w/v) to 50% (w/v) sucrose in 100 mM KCl, 5 mM MgCl_2_, 20 mM Hepes pH 7.4). For high resolution profiles, lysates were loaded onto a 5% (w/v) to 25% (w/v) sucrose gradient (99). Gradients were spun at 35,000 rpm for 1.5 h at 4 °C in a SW55Ti rotor (Beckman) and fractionated using an automated fractionator (Biocomp). RNA fate was detected with a UV lamp at 254 nm.

For ribosome disassembly, lysates were treated with 5 mM EDTA. For puromycin treatment, neurons were incubated with 100 μM PMY for 10 min upon 10 min HRN treatment. For Western blot analysis of puromycylation experiments, cortical neurons were treated either with dimethyl sulfoxide or HRN for 10 min and subsequently with 50 μg/ml of PMY for 10 min. Upon polysome profiling, proteins were isolated from fractions using methanol/chloroform (100).

### Polysome digestion

To digest polysomes, cells were lysed in polysome lysis buffer. An aliquot of the lysate was diluted 1:50 in RNase-free Tris-EDTA buffer. Absorbance at 260 nm was measured and RNase1 amount calculated using the ratio 0.19 U/μl_Lysate_·*A*_260 nm_. Lysates were treated with RNase1 (Thermo Fisher Scientific) for 10 min on ice and subjected to centrifugation.

### Analysis of polysome profiles

Polysome profiles were manually analyzed using the absorbance at 254 nm to identify monosome and polysome peaks. A constant distance between monosomes and polysomes of 8.9 mm was applied. Area under the polysome curves was used as approximation for the number of ribosomes. To analyze polysome rates, the decrease of polysomes were calculated as fold change normalized to CHX control profiles for all time points investigated. An exponential decay kinetics model was used to fit the data points for translation and polysome kinetics (47) (Fig. 1*D*). The least squares method was used for fitting. Polysome rate constants were determined based on these fits.

### Ribosome isolation and mass spectrometry

For ribosome isolation, monosome fraction (fraction 4) and polysomal fractions (fractions 6–8) were used. Polysomal fractions were pooled and diluted in polysome lysis buffer without NP-40 and DOC. Solutions were then centrifuged at 35.000 rpm for 4 h at 4 °C. Supernatant was discarded and pellets resuspended in mass spectrometry lysis buffer (PreOmics). Protein digestion for mass spectrometry analysis was performed according to the manufacturer’s manual (PreOmics). Label-free mass spectrometry was performed as previously described (95).

### Analysis of proteomics data

Mass spectrometry iBAQ quantification values were processed using Bioconductor package DEP (version 1.14.0; https://bioconductor.org/packages/release/bioc/html/DEP.html). After normalization with variance stabilizing normalization, missing values were imputed using DEP::impute with function "mixed" and "knn" for MAR and "zero" for MNAR. Differential abundance was tested with DEP's test_diff wrapper function for limma. Significant proteins were selected using add_rejections with alpha = 0.05 and lfc = log2(1.5). Default parameters were used if not indicated otherwise.

### Transmission electron microscopy

Polysomal fractions from CHX- and HRN-treated cells were pooled and concentrated using concentration tubes (30 kDa filter, Pall) to 50 to 100 μl concentrate. Thereby, buffer was changed several times using the polysome gradient buffer without sucrose. Samples were then collected on formvar coated copper grids (Plano) and stained with Uranyless EM Stain (Electron Microscopy Sciences) for 5 min. Grids were washed three times with double distilled water. Imaging was carried out using the JEOL-1200EX II TEM (JEOL Ltd) at 60 kV. Images were taken using a digital camera (Keen ViewII; Olympus) and processed with the iTEM software package (analySIS Five; Olympus; https://www.olympus-global.com/en/news/2005a/nr050118asfe.html). RNA particle analysis was performed blindly. At least 54 particles (n_CHX,-RNase_ = 54, n_CHX,+RNase_ = 91, n_HRN,-RNase_ = 76, n_HRN,+RNase_ = 65) were randomly selected and measured using ImageJ (https://imagej.net/ij/).

### Lentivirus production and transduction

Rat *EEF2* (NM_017245.2) CDS was cloned into pENTRA1 vector. Lentivirus production was performed as previously described (101). In brief, lentiviral particles were produced in HEK293T cells by cotransfecting the plasmids pVSVG (coding for the vesicular stomatitis virus-glycoprotein), pCMVdR8.91 (expressing gag, pol, and rev genes), and lentiviral expression plasmid. Sixteen hours after transfection, medium was replaced with fresh medium: after 48 h, particles were harvested, concentrated by ultracentrifugation at 24,000 rpm for 2 h. The pellet containing lentiviral particles was resuspended in 1× PBS (supplemented with 5 mM MgCl_2_), and aliquots of the virus were stored at −80 °C until use. The lentiviral titer was determined by infecting HEK cells with different dilutions of the lentivirus and quantifying the number of infected cells after 72 h after the infection. The viral titer was in the range of 10e^9^–10e^11^ transducing units/ml. RCNs were transduced with eEF2-HA or red fluorescent protein expression virus as control for 4 days and subjected to ribosome runoff and polysome profiling.

### Western blotting

For protein analysis, cells were lysed in hot 3× SDS loading buffer. Nucleic acids were digested using Benzonase. Proteins were separated with an SDS-PAGE and transferred to nitrocellulose membrane (pore size 0.2 μm). Primary antibodies (anti-eEF2, anti-p-eEF2, anti-phospho(p)-mTOR, anti-Caspase3, and anti-PARP1 [1:1.000 dilution, all from rabbit, Cell Signaling Technology; # 2332, #2331, #2971, #9662, and #9542]; mouse anti-Rps6, rabbit anti-p-Rps6 [1:1.000 dilution, Cell Signaling Technology; #2317 and #4858]; mouse anti-β-III Tubulin [1:10.000 dilution, Sigma-Aldrich; #T8578]; mouse anti-ACTB [1:5.000 dilution, Sigma-Aldrich; #A2228]; rabbit anti-Rpl7a [1:1.000 dilution, Abcam; #ab70753]; rat anti-HA [1:100 dilution, Helmholtz Center Munich Antibody Core Facility, clone 3F10], mouse anti-PMY [1:5.000 dilution, Sigma-Aldrich; #MABE343]) were diluted in bovine serum albumin blocking solution and incubated with membranes overnight. Membranes were washed and incubated with IRdye labeled secondary anti-mouse, anti-rabbit or anti-rat antibodies (1:10.000 dilution, LI-COR Biosciences). Fluorescence signals were detected with an Odysee scanner (LI-COR Biosciences).

### Statistics

Experiments were performed at least in triplicates. Pharmacological treatments or enzymatic reactions were performed with n ≥ 5 biological replicates. Data are represented as mean ± SEM. *p*-values were calculated using Prism (version 8.0; https://www.graphpad.com/features). No sample was excluded from analysis. For experiments with n > 4 replicates, normal distribution was tested using Kolmogorov-Smirnov test. For multiple comparison, one way ANOVA with subsequent Tukey multiple comparison test was used. For two condition comparison, *p*-values were calculated by using either two-sided paired/unpaired Student’s *t* test or Mann-Whitney U-test. *p* values < 0.05 were considered as statistically significant.

## Data availability

The authors confirm that the data supporting the findings of this study are available within the article and its supplementary materials. The mass spectrometry proteomics data have been deposited to the ProteomeXchange Consortium via the PRIDE (102) partner repository with the dataset identifier PXD043207.

## Supporting information

This article contains supporting information.

## Conflict of interest

The authors declare that they have no conflicts of interest with the contents of this article.
